# Synthesis and Characterization of Bioactive Glass and Zinc Oxide Nanoparticles with Enamel Remineralization and Antimicrobial Capabilities

**DOI:** 10.3390/ma16216878

**Published:** 2023-10-26

**Authors:** Ryota Nagasaki, Keiji Nagano, Takashi Nezu, Masahiro Iijima

**Affiliations:** 1 Division of Orthodontics and Dentofacial Orthopedics, Department of Oral Growth and Development, School of Dentistry, Health Sciences University of Hokkaido, 1757 Kanazawa, Ishikari-Tobetsu 061-0293, Hokkaido, Japan; nagasaki@hoku-iryo-u.ac.jp; 2 Division of Microbiology, Department of Oral Biology, School of Dentistry, Health Sciences University of Hokkaido, 1757 Kanazawa, Ishikari-Tobetsu 061-0293, Hokkaido, Japan; knagano@hoku-iryo-u.ac.jp; 3 Division of Biomaterials and Bioengineering, Department of Oral Rehabilitation, School of Dentistry, Health Sciences University of Hokkaido, 1757 Kanazawa, Ishikari-Tobetsu 061-0293, Hokkaido, Japan; tnezu@hoku-iryo-u.ac.jp

**Keywords:** bioactive glass, zinc oxide, nanoparticle, enamel remineralization, antimicrobial property

## Abstract

Background: We investigated the effect of bioactive glass and zinc oxide nanoparticles on enamel remineralization, as well as their antimicrobial effect on cariogenic microbes. This is the first study that investigated the properties of bioactive glass and zinc oxide nanoparticles with mixed materials. Methods: Fluoride gel (F), bioactive glass microparticles (µB), bioactive glass nanoparticles (nB), zinc oxide nanoparticles (Z), and a mixed suspension of nB and Z (nBZ) were prepared and characterized by scanning and transmission electron microscopy, zeta potential measurement, X-ray diffraction, and acid buffering capacity testing. Further, we performed a remineralization cycle test of 28 days, and nanoindentation testing was carried out during the immersion period, and then the enamel surfaces were examined using scanning electron microscopy. Additionally, the antimicrobial effects of the sample suspensions were evaluated by measuring their minimum microbicidal concentrations against various cariogenic microbes. Results: Our results revealed that nB had a near-circular shape with an amorphous structure and a considerably large specific surface area due to nanoparticulation. Additionally, nB possessed a rapid acid buffering capacity that was comparable to that of μB. In the remineralization test, faster recovery of mechanical properties was observed on the enamel surface immersed in samples containing bioactive glass nanoparticles (nB and nBZ). After remineralization, demineralized enamel immersed in any of the samples showed a rough and porous surface structure covered with mineralized structures. Furthermore, nBZ exhibited a broad antimicrobial spectrum. Conclusions: These results demonstrated that bioactive glass and zinc oxide nanoparticles have superior demineralization-suppressing and remineralization-promoting effects.

## 1. Introduction

The origin of multi-bracket appliances, a fixed orthodontic appliance, dates back to the edgewise appliance designed by Edward H. Angle in 1928 [1]. They have been widely used in orthodontic treatment since then. Multi-bracket appliances are directly attached to the tooth using phosphoric acid-etching treatment on the tooth surface [2], termed the direct bonding method, devised by Newman in 1965 [3]. However, the use of these appliances increases the risk of enamel caries [4,5] and periodontal disease, as wearing the appliance increases plaque stagnation and deteriorates the oral hygiene environment [6]. In addition, the etching treatment causes enamel demineralization. Thus, identifying appropriate measures against these problems is of great clinical significance, and it is crucial to establish good oral hygiene, such as brushing and proper intake of sweets. Furthermore, there is a need to develop functional materials that promote remineralization or suppress demineralization of enamel and determine their application.

In the field of dental healthcare, materials used for caries prevention include fluoride, casein phosphopeptide/amorphous calcium phosphate complex (CPP-ACP), and xylitol, the most common of which is fluoride [5,7]. Fluoride has various methods of application: systemic applications, such as tap-water fluoridation, addition of fluoride to food, and intake of fluoride tablets; and local applications, such as fluoride mouthwash, fluoride-containing dentifrice, and coating the tooth surface with fluoride. However, several studies have reported the risk of fluoride poisoning [8]. Chronic fluoride poisoning caused by long-term excessive intake of fluoride through drinking water or other sources may affect teeth and bones, resulting in fluorosis and osteosclerosis [9]. Additionally, ingesting a large amount of fluoride at once can cause acute poisoning symptoms, such as nausea, vomiting, and abdominal discomfort [8]. Therefore, fluoride should be used with caution, and the application of other caries-prevention materials should be considered.

In the medical field, research on bioactive ceramics has progressed recently, with the range of their applications expanding every year. Among these, bioactive glasses, including 45S5, developed by Larry Hench in 1971 [10], have high biocompatibility and bone conduction capacity [11,12,13], and they have many utilizations; for example, they are used as bone prosthetic materials, dental implants, dentifrice, and in the surface treatment of artificial bones [14,15,16,17,18]. Regarding their use in orthodontic treatment, it is worth noting that when applied to a demineralized enamel surface, bioactive glasses promote remineralization and may contribute to caries prevention [19,20].

Among the functional materials that promote remineralization or possess antimicrobial effects, nanoparticles have been increasingly investigated. They may not only enhance the properties of their bulk material, such as activating chemical reactivity by increasing specific surface area [21,22], change electrical properties by quantum size effect [23,24], and photocatalytic activity and antibacterial properties due to silver and bismuth nanoparticulation [25,26], but may also generate unique properties that material did not originally possess. Among these, the antimicrobial properties of ceramic nanoparticles, such as zinc oxide, are gaining increasing attention [27,28]. With the emergence of drug-resistant microbes because of changing human lifestyles and administering high dosages of drugs during medical treatments, new antimicrobial materials are being developed currently. Antimicrobial ceramic nanoparticles are relatively inexpensive and versatile, as well as safe for use in the human body [29,30], and hence are of interest as a new antimicrobial material.

The remineralization effect of bioactive glass nanoparticles and the antimicrobial effect of zinc oxide nanoparticles have been reported as separate studies. In the present study, we examined the remineralization effect of a composite suspension of bioactive glass and zinc oxide nanoparticles on demineralized enamel surfaces, as well as its antimicrobial effect on cariogenic bacteria, aiming to develop a novel dental material with a superior caries-preventing effect. This is the first study that has investigated the properties of bioactive glass and zinc oxide nanoparticles with mixed materials.

## 2. Materials and Methods

### 2.1. Materials

Bioactive glass microparticles were synthesized using the electric furnace melting method. First, bioactive glass powder mixed with 46.1 mol% SiO_2_, 24.3 mol% Na_2_O, 27.0 mol% CaO, and 2.6 mol% P_2_O_5_ was placed in a platinum crucible, which was then heated and melted at 1550 °C using a high-temperature electric furnace (SSFT-1520, Yamada-Denki Co., Ltd., Tokyo, Japan). The melt was discharged onto a stainless-steel plate with a thickness of 10.0 mm and was rapidly cooled by spreading with a stainless-steel plate of the same thickness. After pre-grinding for 2 min with a sample grinder (TI-200, CMT), it was ground with a fine grinder (NJ-50C, Aishin Nano Technologies Co., Ltd., Saitama, Japan) at a throughput of 60 g/h and a grinding pressure of 1.4 MPa to obtain microparticles (μB) [31].

Bioactive glass nanoparticles, having the same composition as microparticles, were prepared by the sol-gel method according to the procedure described by Hong et al. [32]. After 15.59 mL of tetraethyl orthosilicate (TEOS, 98%, reagent grade, Sigma-Aldrich, St. Louis, MO, USA), 6.71 g of sodium nitrate (≥99.0%, guaranteed reagent grade, Nacalai Tesque, Kyoto, Japan), and 10.35 g of calcium nitrate (99.0%, ACS reagent, Sigma-Aldrich, St. Louis, MO, USA) were dispersed in a liquid mixture comprising 120 mL of deionized water and 60 mL of ethanol (99.5%, reagent grade, Kanto Chemical, Tokyo, Japan); the pH was adjusted to 1 and 2 using citric acid (≥99.0%, extra pure reagent grade, Nacalai Tesque, Kyoto, Japan) while stirring. When the solution became clear, 1500 mL of deionized water containing 1.12 g of dibasic ammonium phosphate (≥99.0%, guaranteed reagent grade, Nacalai Tesque, Kyoto, Japan) was added dropwise to it. During this procedure, the pH was maintained at approximately 11 with aqueous ammonia (28%, guaranteed reagent grade, Nacalai Tesque, Kyoto, Japan). After stirring for 24 h, the precipitate was centrifuged and then washed with deionized water. Subsequently, the precipitate was freeze-dried at −45 °C and 0.01 mbar for 12 h and calcined at 700 °C for 2 h to obtain nanoparticles (nB).

Zinc oxide nanoparticles (Z) were purchased from Sigma-Aldrich (Burlington, MA, USA) (721077). They were dispersed in pure water, and the catalog value of their average particle diameter was ≤40 nm.

### 2.2. Observation of Particle Morphology under Scanning and Transmission Electron Microscopy

The morphology of the nanoparticles was observed using a field emission scanning electron microscope (SEM; JSM-7800F, JEOL Ltd., Tokyo, Japan). The samples were subjected to gold evaporation and observed at an acceleration voltage of 15 kV. For observation using a transmission electron microscope (TEM; JEM-2100F, JEOL Ltd.), a 200-mesh copper grid covered with a collodion support film was used for sample support. Nanoparticles diluted with ethanol were added dropwise onto the grid and dried at room temperature to obtain a sample. The acceleration voltage was set to 200 kV.

### 2.3. Measurement of Specific Surface Area Using the Gas Absorption Method and Evaluation of Surface Electrical Properties through Zeta Potential Measurement

The nitrogen gas adsorption-desorption isotherms of the prepared bioactive glasses (μB and nB) were obtained using an adsorption measurement device (Autosorb 6AG, Yuasa Ionics Co., Ltd., Osaka, Japan). Prior to each measurement, the samples were degassed at 200 °C under reduced pressure (10^−3^ Torr) for 15 h. Adsorption and desorption operations were performed by cooling to −196 °C with liquid nitrogen. Based on the obtained isotherms, Brunauer–Emmett–Teller (BET) analysis was performed to calculate the specific surface areas [33], and the sizes of µB and nB particles were compared.

To evaluate the surface electrical properties of nanoparticles, the zeta potential was measured by the laser-Doppler electrophoresis method using a zeta-potential measurement device (Delsa Nano HC, Beckman Coulter Inc., Brea, CA, USA). Measurements were conducted in deionized water at 25 °C and pH 7. The zeta potential was calculated based on the following Smoluchowski equation [34]:

ζ = Uη/ε(1)

where ζ is the zeta potential, U is the electrophoretic mobility, η is the viscosity, and ε is the permittivity of the medium.

### 2.4. Crystal Structure Analysis by X-ray Diffraction

The crystal structure of each bioactive glass particle sample (μB and nB) was analyzed by X-ray diffraction (XRD; Rint2500, Rigaku Corporation, Tokyo, Japan). The analysis was conducted using the concentration method with an X-ray tube voltage of 40 kV, a tube current of 100 mA, a scanning range of 10–60°, and a scanning speed of 0.15°/min.

### 2.5. Evaluation of Acid Buffering Capacity

The μB and nB particles were immersed in 5 mL of an acetic acid aqueous solution (pH 4.5) at various concentrations (0.1, 0.5, 1, 5, 10, and 50 mg/mL), and the pH was measured over time (5, 15, 30, 45, and 60 min) using a pH meter (F-72, HORIBA, Kyoto, Japan) to evaluate their acid buffering capacity. The pH of the acetic acid aqueous solution alone was considered a control.

Further, the acid buffering capacities of the particle samples were compared. Based on the results of the acid buffering capacity test on nB, the concentrations of nB and µB samples were set at 5.0 mg/mL. In addition, the concentration of zinc oxide nanoparticles was set at 200 µg/mL based on the study of Almoudi et al., which evaluated the antimicrobial effect of the particles (*n* = 3) [35].

### 2.6. Evaluating the Capacity of the Particles to Induce Enamel Remineralization

Using a low-speed rotary cutting machine (IsoMet, Buehler, Lake Bluff, IL, USA; equipped with a diamond blade), blocks of enamel were cut out from extracted human teeth at low speed under water cooling and embedded in epoxy resin (Epofix, Struers, Ballerup, Denmark). After coarse polishing with waterproof abrasive papers #240 and #600 using a small surface polishing machine (ML110N, Maruto, Tokyo, Japan), the embedded samples were mirror polished using diamond abrasive materials of 3 µm diameter and then of 1 µm diameter; they were further polished by aluminum oxide abrasive materials of 0.3 µm diameter. The samples were then fixed in a sample holder for the nanoindentation test.

**Figure 1:**
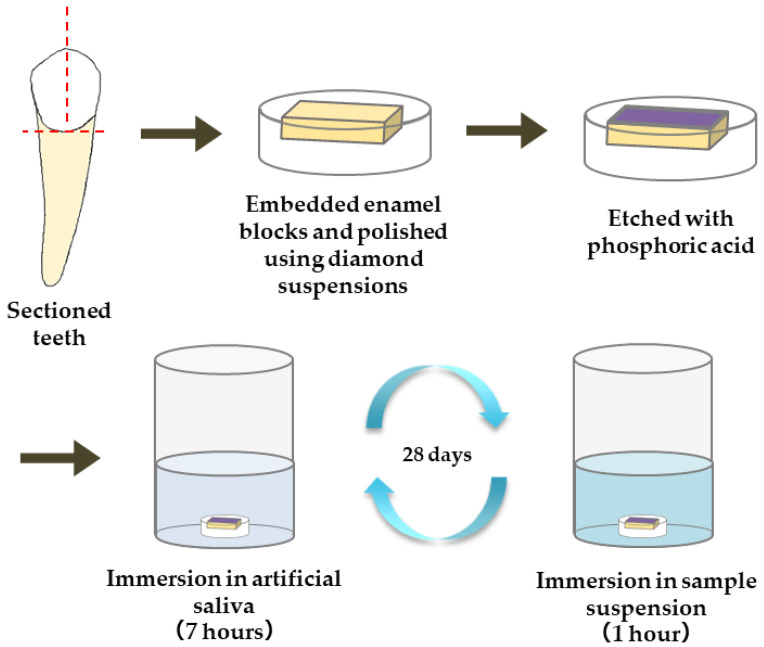
Procedure for preparation of enamel samples and remineralization test.

The materials used for the remineralization test are summarized in Table 1. The following five materials were applied to the enamel samples: commercially available gel containing sodium fluoride (1450 ppm)/cetylpyridinium chloride (Check-up gel Mint, Lion Corporation, Tokyo, Japan) (F), µB, nB, and nB+Z (nBZ) (Figure 1). The surface of the enamel samples was demineralized by etching treatment (15 s) with 35% phosphoric acid (3 M; Transbond XT Etching Gel, Monrovia, CA, USA). Then, a remineralization cycle test was performed for 28 days, alternating between 7-h immersion in a remineralization solution (pH 6.8) and 1-h immersion in the suspension of each test material. The concentration of the suspensions was adjusted with deionized water to 5.0 mg/mL for nB and µB and 200 µg/mL for Z. The remineralization solution used was artificial saliva adjusted to have a Ca/PO_4_ ratio of 1.67 by adding CaCl_2_ and NaH_2_PO_4_ to deionized water. CH_3_COOH and NaOH were used for pH adjustment. A sample immersed only in the remineralization solution was used as a control (Con).

The nanoindentation test was performed on enamel samples before and after demineralization as well as after the remineralization test (1, 7, and 28 days) to evaluate the mechanical properties of the enamel samples (hardness and elastic coefficient). A Berkovich-type diamond indenter (a triangular pyramid with a ridge line angle of 115°) was used for the measurement. To investigate the mechanical properties of the demineralized surface and deep layers, the indentation load was set to the following two conditions: 10 mN (indentation depth of approximately 400 nm) and 100 mN (indentation depth of approximately 1200 nm). Five enamel samples were prepared for each material, and each enamel sample was examined at five sites in four fields of view.

After critical point drying and gold evaporation, the enamel samples were observed by SEM before and after demineralization, as well as after 28 days of the remineralization test.

### 2.7. Evaluation of Antimicrobial Properties

In the antimicrobial test, the minimum microbicidal concentration (MMC) of each test material was measured using the liquid microdilution method in a 96-well plate. The test examined the four materials used in the remineralization test, and a 10% diluted solution prepared from each material was used. Table 2 lists the 10 microbial species selected as test microbes. The microbial cultures were adjusted to a turbidity of OD_600nm_ = 1.0 × 10^−3^ with BHI (brain heart infusion) agar, and 100 µL of each microbial solution was inoculated onto a plate. Then, 3 µL of the culture solution was inoculated on fresh BHI agar medium, and MMC was evaluated by the presence or absence of microbial growth (*n* = 3).

### 2.8. Statistical Analysis

Statistical analysis was performed using statistical analysis software (SPSS Statistics 25, IBM). A one-way analysis of variance was used to test the differences in the mean values of the test materials, and Tukey’s test was used for subsequent multiple comparisons. *p* < 0.05 was considered statistically significant.

## 3. Results

### 3.1. Morphological Observation of Particle Samples and Identification of Their Crystal Structures

**Figure 2:**
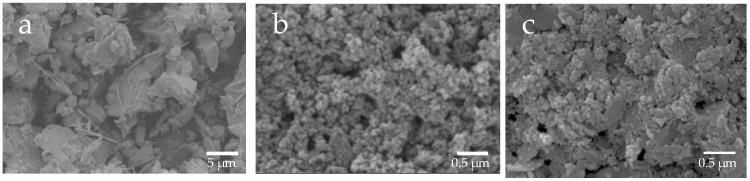
Representative SEM photomicrographs of the sample particles ((**a**) 3000× magnification image of µB; (**b**) 30,000× magnification image of nB; (**c**) 30,000× magnification image of Z).

**Figure 3:**
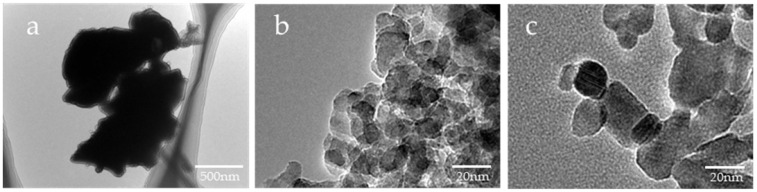
Representative TEM photomicrographs of the sample particles ((**a**) 10,000× magnification image of µB; (**b**) 200,000× magnification image of nB; (**c**) 200,000× magnification image of Z).

Based on the SEM images, the shape of µB was irregular, having rounded and angular shapes, and its particle diameter was uneven, ranging from several micrometers to several tens of micrometers (Figure 2). The particles of nB and Z were found to have a near-circular shape, and each particle exhibited agglomeration. Moreover, as determined by the high-magnification TEM images, the particle diameters of nB and Z were approximately 20 nm and 40 nm or shorter, respectively (Figure 3), and the particle diameter of Z was comparable to its catalog value.

**Figure 4:**
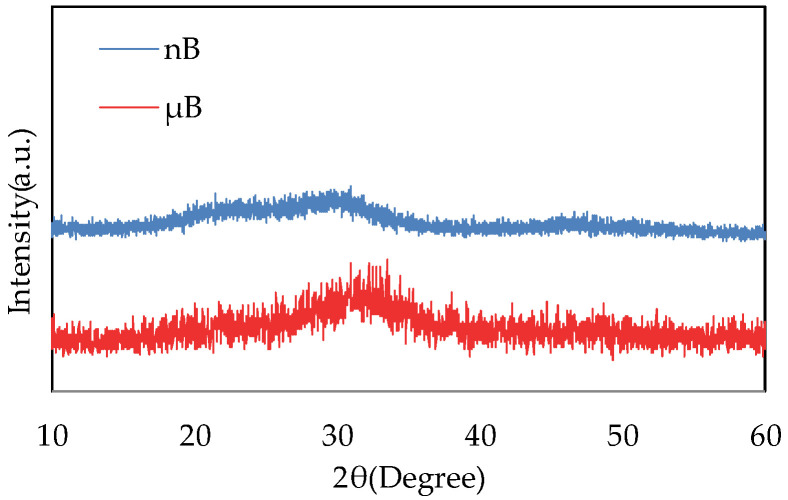
XRD patterns of nB and µB.

The XRD patterns of µB and nB show no sharp peaks, indicating that they have an amorphous structure (Figure 4).

### 3.2. Measurement of the Specific Surface Area of Each Particle Sample and Evaluation of Surface Charge Properties by Zeta Potential

**Figure 5:**
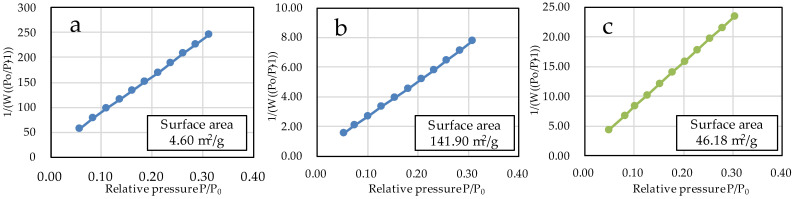
BET plots of the sample particles ((**a**) µB; (**b**) nB; (**c**) Z).

**Figure 6:**
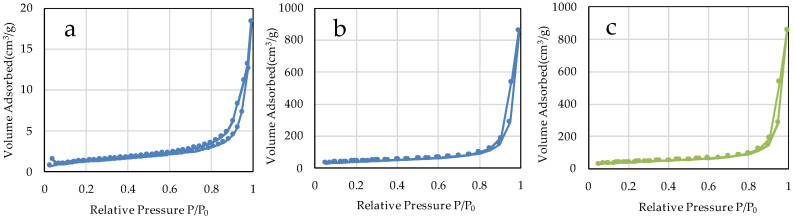
N_2_ adsorption-desorption isotherms of the sample particles. ((**a**) µB; (**b**) nB; (**c**) Z).

The specific surface areas of µB and nB calculated by the multipoint BET method were 4.60 m^2^/g and 141.90 m^2^/g, respectively (Figure 5), showing a marked increase in the specific surface area due to the nanosizing of the particles. The specific surface area of Z was 46.18 m^2^/g. The adsorption/desorption isotherms with nitrogen gas show the presence of hysteresis in all specimens and exhibit the H1 type of IUPAC classification (Figure 6) [36].

**Figure 7:**
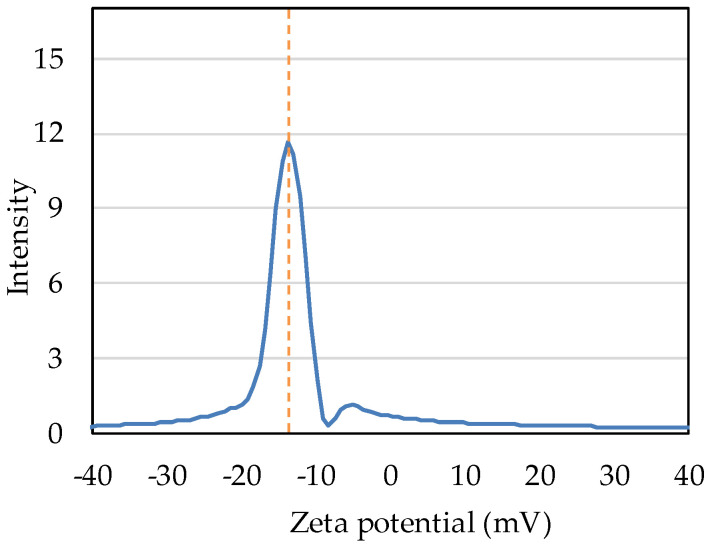
Intensity distribution of the zeta potential of nB.

Intensity distribution measurement shows that the zeta potential of nB in deionized water was −13.71 mV (Figure 7).

### 3.3. Evaluation of Acid Buffering Capacity

**Figure 8:**
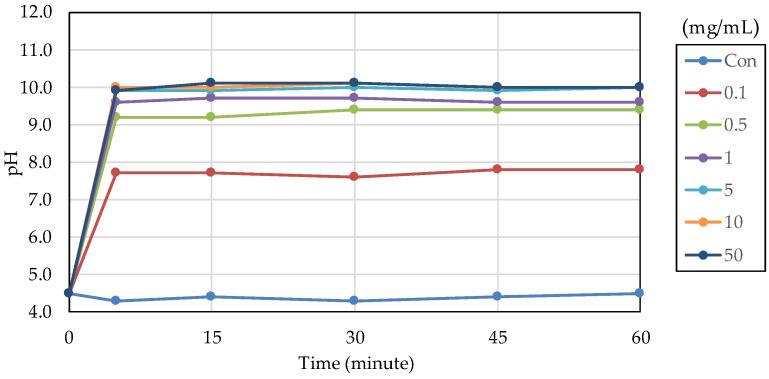
Changes in the pH of acetic acid solutions in which nB were immersed.

**Figure 9:**
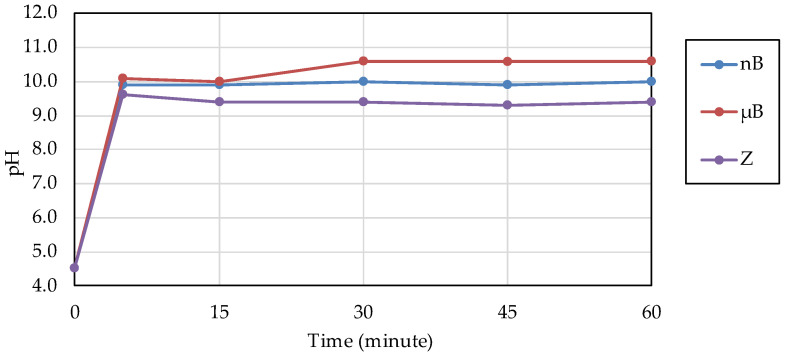
Changes in the pH of acetic acid solutions in which the sample particles were immersed.

The acid buffering capacity of nB was tested at different concentrations. At any concentration, the pH increased rapidly after the start of immersion, and at concentrations of 5.0 mg/mL and higher, the pH reached approximately 10 after 5 min of immersion (Figure 8). Then, the acid buffering capacities of the particle samples were compared. Like nB, both µB and Z show a rapid increase in pH after immersion, and the pH reached 9.5 or higher after 5 min of immersion of any particle sample (Figure 9). There was no size-dependent difference between µB and nB.

### 3.4. Evaluation of Enamel Remineralization Capacity

**Figure 10:**
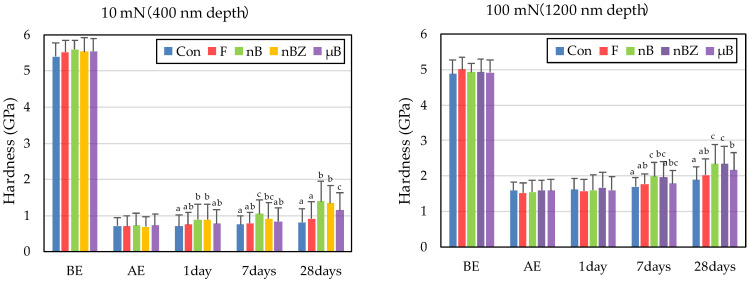
Mean hardness values of enamel surfaces before and after etching and after remineralization tests (BE, before etching; AE, after etching; 1 day, 1-day immersion; 7 days, 7-day immersion; 28 days, 28-day immersion. Identical letters indicate that mean values are not significantly different (*p* < 0.05, Tukey test)).

**Figure 11:**
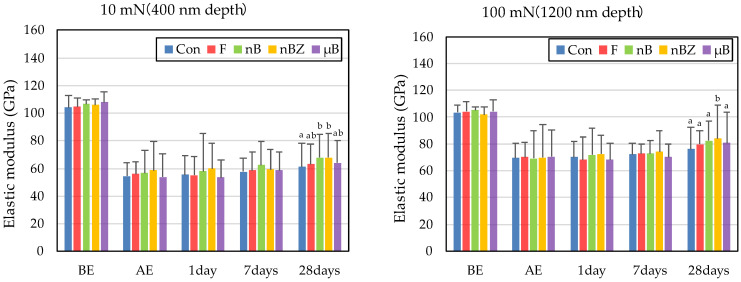
Mean elastic modulus values of enamel surfaces before and after etching and after remineralization tests (BE, before etching; AE, after etching; 1 day, 1-day immersion; 7 days, 7-day immersion; 28 days, 28-day immersion. Identical letters indicate that mean values are not significantly different (*p* < 0.05, Tukey test)).

The etching treatment greatly reduced the hardness and elastic coefficient of the surface of all enamel samples (Figure 10 and Figure 11). This decrease in the mechanical properties of the enamel surface was more prominent when tested at a load of 10 mN than at a load of 100 mN in any sample. In the subsequent remineralization test, the mechanical properties of the enamel surface gradually recovered with the progression of the immersion period (1, 7, and 28 days).

After 28-day immersion, the enamel samples immersed in nB show significant recovery in hardness compared with those immersed in µB when tested at a load of 10 mN or 100 mN. Like hardness, the elastic coefficient was greater in the enamel samples immersed in nB after the remineralization test than in those immersed in µB. However, there was no significant difference between the two groups, except for the significantly high recovery observed in samples immersed in nBZ after 28 days at 100 mN load.

Compared with the enamel samples immersed in F, those immersed in the materials containing bioactive glass nanoparticles (nB and nBZ) show significant recovery in hardness after 28-day immersion when tested at a load of 10 mN or 100 mN. Similarly, the enamel samples immersed in nB or nBZ had a greater elastic coefficient after the remineralization test than those immersed in F. However, there was no significant difference between the two groups, except for the significantly high recovery observed in the samples immersed in nBZ after 28 days at 100 mN load.

**Figure 12:**
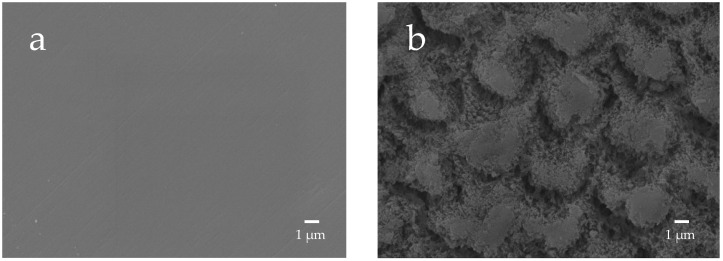
Representative SEM photomicrographs of the enamel surface before and after demineralization ((**a**), before etching; (**b**) after eching; 5000× magnification image).

**Figure 13:**
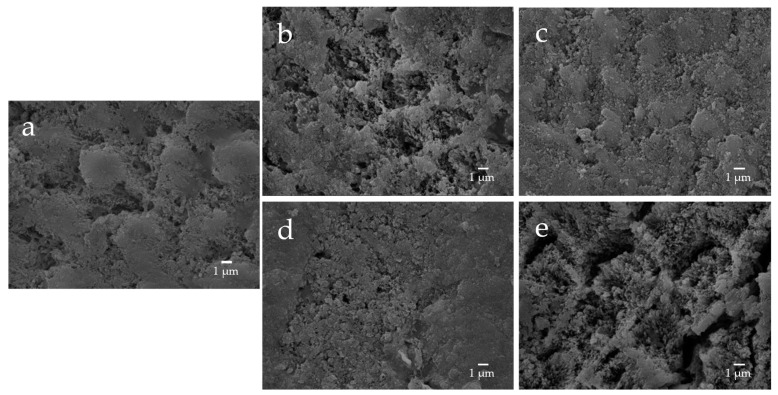
Representative SEM photomicrographs of the enamel specimens after remineralization tests for 28 days ((**a**) control; (**b**) F; (**c**) nB; (**d**) nBZ; (**e**) µB; 5000× magnification image).

The enamel surface before etching (BE) shows a smooth and uniform surface structure, whereas the enamel surface after etching treatment (AE) shows a rough and porous honeycomb-shaped surface structure (Figure 12). The SEM images of the surface of the enamel samples immersed in different materials revealed that after 28-day immersion, the porous structure on the surface of all enamel samples was covered with remineralized matter-like structures derived from fluoride and bioactive glass (Figure 13). The demineralized surface of the control sample (Con), which was immersed only in the remineralization solution, exhibits mineralized matter-like structures because of the action of minerals in the remineralization solution. The surface of enamel samples immersed in F and µB retains several enamel demineralization structures formed by the etching treatment, whereas the surface of enamel samples immersed in materials containing bioactive glass nanoparticles (nB and nBZ) shows denser deposits of mineralized matter and a porous honeycomb-like surface structure disappeared.

### 3.5. Evaluation of Antimicrobial Properties

**Figure 14:**
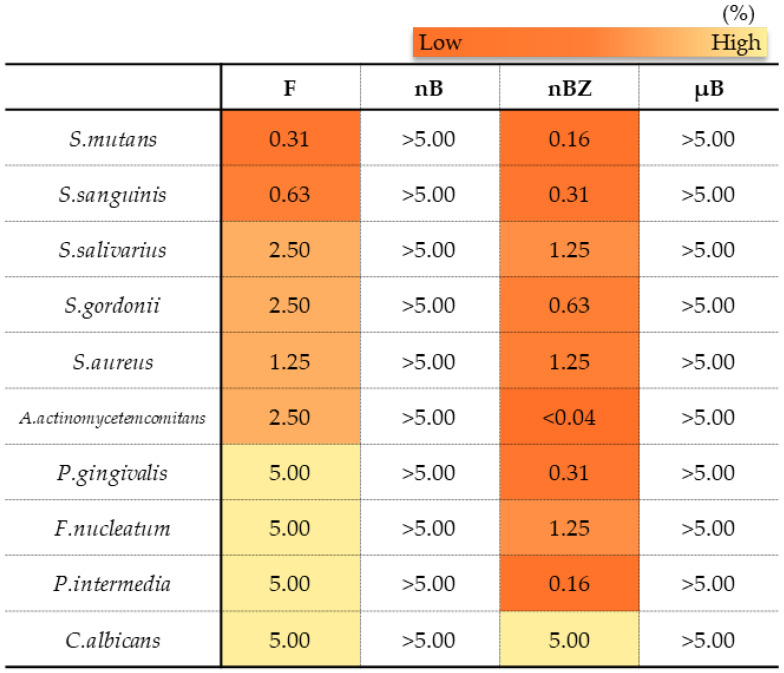
Minimum microbicidal concentration (MMC) ranges of sample suspensions.

In the antimicrobial test, F and nBZ were found to have a low MMC against any microbes tested, including gram-positive bacteria, gram-negative bacteria, and the fungus *Candida albicans*, showing a broad antimicrobial spectrum (Figure 14). Other particle samples show no antimicrobial effect when tested in a diluted suspension at a concentration of 5% or lower, as used in this study.

## 4. Discussion

### 4.1. Properties of Bioactive Glass Nanoparticles

We prepared bioactive glass particles using two methods: the electric furnace melting method and the sol-gel method. In general, the electric furnace melting method, used to prepare microparticles, is classified as a breakdown process, whereas the sol-gel method, used to prepare nanoparticles, is classified as a build-up process. In the breakdown process, large lumps are pulverized to a desired size by mechanical grinding or other techniques, and the diameter of the resulting particles ranges from several microns to sub-micron meters. On the other hand, the build-up process constructs particles through particle growth from the core at the atomic or molecular level using chemical and other reactions, and the diameter of the resulting particles is mainly in sub-microns or smaller. In the sol-gel method, the hydrolysis and polycondensation of TEOS in a mixed solvent of deionized water and ethanol induce the formation of primary colloidal nanoparticles (sol), followed by the preparation of wet gels by the three-dimensional and advanced growth of silica networks in acid solvents and the formation of secondary particles in basic solvents [37]. The bioactive glass obtained using the acid-basic solvent has a large number of gaps and thus has greatly enhanced porosity compared with the bioactive glass derived from the electric furnace melting method [38,39]. In the present study, the specific surface areas of microparticles and nanoparticles determined by the BET method were 4.60 m^2^/g and 141.90 m^2^/g, respectively, which suggests that the nanosizing of the particles and improvement in porosity greatly increased the specific surface area, leading to improved chemical reactivity. However, the adsorption/desorption isotherm with nitrogen gas shows an H1-type hysteresis pattern, suggesting that each particle forms an aggregate (Figure 6). This is consistent with finding in the SEM and TEM images.

Zeta potential is defined as the potential of the “slide plane” at which liquid flow begins to occur in the electric double layer formed around particles in a solution, and as it approaches zero, the mutual repulsive force between particles weakens, resulting in the aggregation of particles. Therefore, zeta potential is used as an index for the evaluation of dispersion stability. Gumusutas et al. reported that particles completely aggregate when the absolute value of the zeta potential is below 5 mV and that monodispersity can be ensured at 30 mV or higher [40]. The zeta potential of nB prepared in the present study was −13.71 mV. This suggests that nB, when prepared by the method used in this study, has moderate dispersion stability that results in neither complete aggregation nor monodispersity. To ensure further dispersion stability, a method for preparing particles with an absolute zeta potential exceeding 30 mV is required.

Based on the XRD patterns of the particle samples, both nB and µB were found to have an amorphous structure. The bioactivity of a bioactive glass decreases as its crystallinity increases [15], and having an amorphous structure is considered essential for a bioactive glass to exhibit high bioactivity.

In an aqueous environment, a rapid exchange of sodium ions, derived from bioactive glass particles, with hydrogen ions occurs, resulting in an increase in pH. This increase in pH precipitates excess calcium and phosphate ions supplied by the bioactive glass, forming a calcium phosphate layer, which then crystallizes into a hydroxycarbonate apatite layer [41]. Our acid buffering capacity test revealed a rapid increase in pH within 5 min after immersion of nB or µB, but there was no size-dependent difference, suggesting that nB and µB had a comparable demineralization-suppressing effect.

### 4.2. Evaluation of Remineralization by Changes in Mechanical Properties of the Surface of Enamel Samples

In the nanoindentation test, the indentation depths at the load of 10 mN and 100 mN were approximately 400 nm and 1200 nm, respectively, and the indentation widths at this time were approximately 1–3 µm and 15 µm, respectively. In contrast, the demineralization depth of enamel by phosphoric acid etching is approximately 8–10 µm [42], and the diameter of the enamel rod is thought to be approximately 4 µm. Therefore, testing at the load of 10 mN measures the enamel rod alone, whereas testing at the load of 100 mN measures the area covering not only the enamel rod but also the enamel rod sheath, which is thought to have a high composition ratio of organic matter, likely leading to properties closer to the bulk material.

To quantify the examined mechanical properties, the rates (%) of recovery in hardness and elastic coefficient were determined from the results obtained with an indentation load of 100 mN (difference between BE and after 28-day immersion). The rates (%) of recovery in hardness (H) and elastic coefficient (E) were calculated based on the following equations [43]:

(2) $$H_{\mathrm{Recover}}\;=\frac{\left(H_{28D}-H_{AE}\right)}{\left(H_{BE}-H_{AE}\right)}\;\times \;100\;(\%);\;E_{\mathrm{Recover}}\;=\frac{\left(E_{28D}-E_{AE}\right)}{\left(E_{BE}-E_{AE}\right)}\;\times \;100\;(\%)$$

The rates of recovery in hardness and elastic coefficient were 9.0% and 20.1%, respectively, in Con; 14.5% and 28.4%, respectively, in F; 23.5% and 36.1%, respectively, in nB; 22.2% and 44.4%, respectively, in nBZ; and 17.1% and 30.7%, respectively, in µB. Thus, the materials containing bioactive glass nanoparticles (nB and nBZ) tended to have higher recovery rates than Con or F.

The remineralization test shows that after 28-day immersion, all samples, except F or µB, caused the enamel surface to be covered in mineralized structures derived from the sample suspensions; the surface of enamel immersed in F or µB still exhibits the porous structure observed after demineralization (Figure 13). Meanwhile, the surface of enamel subjected to remineralization by bioactive glass nanoparticles (nB and nBZ) shows a greater loss of the porous structure. This was likely because small apatite-like crystal structures generated by bioactive glass nanoparticles and the remineralization solution were densely deposited on the demineralized enamel surface and deposited in the pores after demineralization. These suggested that bioactive glass nanoparticles promote remineralization of enamel to a higher degree via the remineralization solution.

However, damages to the enamel surface caused by the phosphoric acid-etching treatment were prominent, and the mechanical properties of the enamel surface after 28 days of immersion were still inferior to those of the pre-demineralization enamel. In the present study, we performed a remineralization test that assumed demineralized enamel due to etching around the multi-bracket appliance. However, a remineralization cycle that assumes an early carious lesion initiated by a slight dissolution of only the enamel surface layer may yield different results [44]. To generate a remineralized layer with a higher quality, concentrations of test suspensions and longer immersion tests may need to be considered.

Comparing the recovery of mechanical properties (hardness and elastic coefficient), nB (23.5% and 36.1%, respectively) exhibits superior remineralization potential than µB (17.1% and 30.7%, respectively). In addition, after 28 days of the remineralization test, the enamel surface immersed in nB showed significant recovery in hardness compared with that immersed in µB, indicating size-dependent enhancement of the effect, as reported by a previous study [45]. Although physical blockage of the honeycomb-shaped demineralized enamel surface by smaller particles may have affected the improvement in hardness [41], the present study found no clear basis for this significant recovery of the mechanical properties of enamel immersed in nB. Thus, a more detailed structural evaluation of mineralized matter-like deposits at the crystal level is needed in the future.

### 4.3. Antimicrobial Effect of Nanoparticle Suspensions

Many studies report that the formation of zinc oxide into nanoparticles can increase antimicrobial activity [46]. This study also showed that nBZ, containing zinc oxide nanoparticles, had an MMC comparable to that of the control drug F, which contained cetylpyridinium chloride, and they showed a broad antimicrobial spectrum covering gram-positive bacteria such as the cariogenic bacteria *Streptococcus mutans*; gram-negative bacteria such as periodontopathic bacteria; and the fungus *Candida albicans*. In addition, MMC by zinc oxide nanoparticles under the conditions in this study was basically consistent with the report from Almoudi et al. [33]. These results strongly indicate that the zinc oxide nanoparticles maintain oral hygiene in good conditions.

nB was also expected to exhibit antimicrobial activity because a bioactive glass generally showed substantial antimicrobial activity [47]. However, we did not detect its antimicrobial activity in any microbes in our experimental conditions.

Although the action mechanism of zinc oxide nanoparticles remains unclear, several theories have been proposed, such as the generation of reactive oxygen species (ROS) [48], the release of antimicrobial ions (Zn^2+^) [49], and inhibition of metabolism through interactions between particles and cell membranes [50]. It is well known that zinc oxide exhibits antimicrobial activity even when its powder form is added to a material, and zinc oxide powders are widely used in toothpaste and mouthwash [51]. However, the powder condition of zinc oxide does not seem to show ROS generation or metabolic inhibition. This might be the reason why the antimicrobial activity of zinc oxide increases when it becomes a nanoparticle. Although these effects ultimately lead to the morphological disruption of microorganisms, the detailed mechanism is unknown.

Since the mechanisms of these theories are centered on damage to the bacterial cell membrane, they are not likely to result in the development of drug resistance in bacteria [52]. In the present study, zinc oxide nanoparticles showed a rapid acid buffering capacity in addition to an antimicrobial effect. This is likely because the following chemical reaction occurred in the acetic acid solution:

ZnO + 2H^+^ → Zn^2+^ + H_2_O (3)

Therefore, zinc oxide nanoparticles may not only be effective as an alternative antimicrobial drug against oral microbes, such as cariogenic bacteria, but may also possess a demineralization-suppressing effect.

### 4.4. Future Clinical Application of Nanoparticle Materials

Developing a new gel is crucial for the clinical application of nanoparticle materials in the future. A new gel that can be applied after tooth brushing with regular dentifrice can be used in more focused care for patients who are at high risk of caries, such as those with tooth eruptions, those receiving orthodontic treatment, and those with exposed root surfaces. Furthermore, with reference to the local application of fluoride with a mouthpiece, as demonstrated in the study by Englander et al. [53], we believe that the application of such a new gel to the inner surface of a mouthpiece-type orthodontic appliance will be effective. The present study immersed enamel samples in a suspension three times for 24 h in a remineralization cycle, assuming that a mouthpiece is removed during meals in the morning, noon, and night and that a gel is applied again before putting on the mouthpiece.

Mouthpiece-type orthodontic appliances include those used for retention management to prevent relapse upon completion of active treatment with multi-bracket appliances and those used for active treatment. However, the prevalence of white spots during active treatment with multi-bracket appliances is reported to be 68.4% [54], and these appliances are expected to contribute to a remineralization-promoting effect on the demineralized surface. When used for active treatment, it has been reported that wearing a mouthpiece-type orthodontic appliance inhibits the self-cleaning effect of saliva, acid buffering capacity, and remineralization effect and that there is a risk of acid erosion caused by drinking highly acidic cariogenic beverages (such as refreshing and sports beverages) when wearing an appliance, as the beverages remain on the inner surface of a mouthpiece [55]. Thus, the materials examined in the present study are expected to exert a caries-preventing effect. Since the nanoparticles used in this study showed aggregation of primary particles, it is necessary to consider stabilizing the particles by using a capping agent as a future investigation [56]. The gel applied to the inside of the mouthpiece must maintain its viscosity as a gel so that it can stagnate for a certain period of time and achieve a sustained effect. In addition, because the increase in viscosity may decrease the permeability to the enamel surface, the concentration also needs to be re-examined. It is necessary to examine whether the concentration combines sufficient biological safety for eventual clinical application.

## 5. Conclusions

The results of this study can be summarized as follows:

- The particle diameter of bioactive glass microparticles was uneven, ranging from several micrometers to several tens of micrometers. On the other hand, the bioactive nanoparticles were agglomerated, but had individual particle diameters of approximately 20 nm.
- Bioactive glass nanoparticles and microparticles possess rapid acid buffering capacity.
- The enamel samples immersed in the materials containing bioactive glass nanoparticles (nB and nBZ) exhibit superior recovery of mechanical properties compared with those immersed in other materials, and apatite-like structures are deposited on the surface of these enamel samples.
- Materials containing zinc oxide nanoparticles (nBZ) have a broad antimicrobial spectrum.
- Bioactive glass and zinc oxide nanoparticles can suppress demineralization through acid buffering and antimicrobial effects on cariogenic bacteria while promoting remineralization.

## Figures and Tables

**Table 1:** The list of particle samples and their composition used in the study.

Group	Samples
Con	only artificial saliva
F	fluoride + cetylpyridinium chloride
nB	bioactive glass nanoparticle
nBZ	bioactive glass nanoparticle + zinc oxide nanoparticle
μB	bioactive glass microparticle

**Table 2:** List of bacteria used in antimicrobial testing.

	Species	Strain
Gram positive	*Streptococcus mutans*	NTCT 10499
	*Streptococcus sanguinis*	JICC 136
	*Streptococcus salivarius*	HHT
	*Streptococcus gordonii*	DL-1
	*Staphylococcus aureus*	JCM 20624
Gram negative	*Aggregatibacter actinomycetemcomitans*	Y4
	*Porphyromonas gingivalis*	ATCC 33277
	*Fusobacterium nucleatum*	ATCC 25586
	*Prevotella intermedia*	JCM 8353
Fungus	*Candida albicancs*	FC18

## Data Availability

Not applicable.
